# Cortical Laminar Bone Membrane: Transforming Regenerative Approaches in Dentistry

**DOI:** 10.7759/cureus.75138

**Published:** 2024-12-05

**Authors:** Rohida Nehal, Ayush Agrawal, Vinayak Thorat, Sanpreet S Sachdev

**Affiliations:** 1 Periodontology, Bharati Vidyapeeth (Deemed to be University) Dental College and Hospital, Navi Mumbai, IND; 2 Oral Pathology and Microbiology, Bharati Vidyapeeth (Deemed to be University) Dental College and Hospital, Navi Mumbai, IND

**Keywords:** acceleration of bone formation, alveolar bone loss, alveolar bone resorption, generalized periodontitis, osteogenesis, osteoinduction

## Abstract

Cortical laminar bone membrane (CLBM) is well known for its extraordinary mechanical properties, biocompatibility, and osteoconductive potential, and thus, it has been revealed as a revolutionary biomaterial in periodontal and alveolar bone regeneration. CLBM offers a superior alternative to traditional barrier membranes used in guided bone regeneration (GBR) and guided tissue regeneration (GTR). CLBM represents a significant advancement in managing complex defects by overcoming common limitations such as premature degradation and inadequate soft tissue support. The review combines insights from current research to evaluate the properties, biological mechanisms, and clinical applications of CLBM, alongside its comparative advantages and limitations.

## Introduction and background

Bone defects can be caused by numerous reasons; the most significant ones in dental medicine include periodontal disease, trauma, peri-implantitis, and atrophy. In these cases, bone loss not only affects the esthetic appearance but also diminishes the structural integrity and function of the dental and facial framework. In order to overcome these issues, the demand for advanced treatment is increasing, and the search for innovative materials and techniques to facilitate bone regeneration has become extremely important [1,2].

Guided bone regeneration (GBR) and guided tissue regeneration (GTR) have been recognized as gold standards among the numerous techniques employed. These methods use barrier membranes in a strategic manner to create a protective environment that prevents the infiltration of unwanted cell populations such as fibroblasts and epithelial cells while also supporting osteogenesis [1,3,4]. However, traditional membranes, including polytetrafluoroethylene (PTFE) and collagen-based alternatives, are often limited by insufficient mechanical strength, a high risk of soft tissue invasion, and in some cases, the need for surgical removal on non-resorbable variants [2,5]. The introduction of the cortical laminar bone membrane (CLBM), derived from xenogeneic bone tissue has revolutionized regenerative dentistry. CLBM's unique properties, including high mechanical stability, controlled degradation, and osteoconductivity, enable it to overcome the limitations of traditional membranes. Particularly, in the context of large or complex defects, CLBM has demonstrated remarkable clinical outcomes. The present review probes into the structural characteristics, biological mechanism, clinical applications, and challenges associated with CLBM supported by evidence from recent studies.

## Review

Key properties of cortical laminar bone membrane

CLBM is characterized by its rigidity, which significantly surpasses the mechanical strength of traditional collagen membranes. This rigidity ensures that the membrane effectively maintains space within bone defects, preventing collapse under soft tissue pressure. This property is critical when regenerating extensive or irregular defects, where maintaining structural integrity is essential for predictable outcomes [6-8]. The integrity of the membrane is maintained by adapting it onto the site and fixing it in place using stainless steel mini screws [6]. A study by Rossi et al. highlights the use of CLBM in cases of severe bone atrophy [7]. The ability of the membrane to stabilize graft materials and maintain defect morphology was instrumental in achieving successful regeneration. Such mechanical stability is particularly important in demanding clinical scenarios, such as lateral ridge augmentation or large vertical defects, where traditional membranes often fail to provide adequate support [9]. Since CLBM is derived from xenogeneic bone, it undergoes stringent processing to eliminate antigenic proteins while preserving its osteoconductive properties. This ensures compatibility with human tissues and creates an optimal surface for osteoblast attachment and mesenchymal stem cell differentiation [1,4,10]. Tumedei et al. emphasized that CLBM not only supports the adhesion and proliferation of osteogenic cells but also facilitates their integration into the host bone tissue. This compatibility with surrounding tissues is a crucial factor in achieving long-term stability and functionality in regenerated bone [5]. Unlike rapidly degrading collagen membranes, CLBM has a much slower and more controlled resorption profile. This property ensures that the membrane provides prolonged protection against soft tissue invasion while supporting the healing process over an extended period [8,10]. Schuh et al. investigated the use of CLBM in multilayer regenerative techniques and found that its longevity significantly improved outcomes, particularly in esthetically demanding areas. The controlled degradation rate allows for sufficient time for bone remodeling, thus reducing the chances of complications such as soft tissue intrusion or incomplete regeneration [8].

Biological mechanism of action

**Figure 1:**
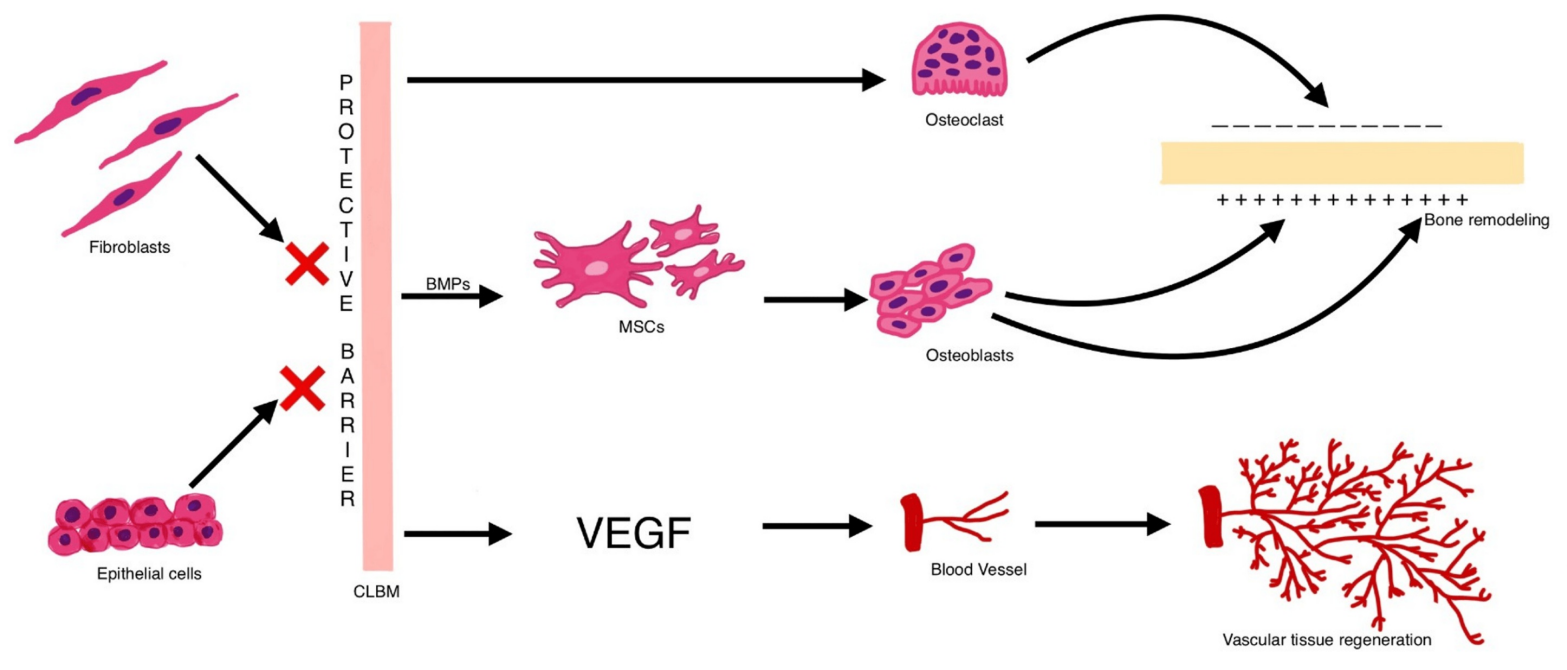
Biological mechanisms of action of cortical laminar bone membrane BMP: bone morphogenic protein, MSCs: mesenchymal stem cells, VEGF: vascular endothelial bone factor Image credit: Nehal Rohida, Ayush Agrawal

CLBM acts through various mechanisms to induce bone formation (Figure 1). The manner in which bone is laid down following the use of CLBM differs from other membranes, wherein unlike the latter, it is similar to natural bone remodeling.

Osteogenesis Support

CLBM functions as a scaffold that mimics the natural extracellular environment of bone. Its surface promotes the adhesion of osteogenic cells, facilitating the migration and differentiation of mesenchymal stem cells into osteoblasts. This process is essential for the formation of new bone and the eventual remodeling of the defect site [5,6,10]. Furthermore, the membrane supports osteoclastic activity, which is vital for remodeling and integration with the host bone. This dual support for osteogenesis and remodeling distinguishes CLBM from purely barrier-focused materials, such as PTFE membranes.

Growth Factor Modulation

CLBM acts as a reservoir for bioactive molecules, including bone morphogenetic proteins (BMPs) and vascular endothelial growth factor (VEGF). BMPs are critical for osteogenesis, while VEGF stimulates angiogenesis, ensuring the delivery of oxygen and nutrients to the regenerating site [3,5]. Studies have shown that the synergistic effects of BMPs and VEGF enhance both bone and vascular tissue regeneration, making CLBM particularly effective in complex defect management. For example, Lee and Kim demonstrated how CLBM's ability to modulate these growth factors contributed to accelerated healing and improved clinical outcomes [3].

Barrier Effectiveness

The dense structure of CLBM prevents the infiltration of epithelial and fibroblastic cells into the defect site. This barrier function is crucial in maintaining an undisturbed environment for bone regeneration, particularly in GTR procedures where the regeneration of periodontal ligament and connective tissue is prioritized [1,9].

Clinical applications

CLBM has proven effective in treating both vertical and horizontal periodontal bone defects. Recent studies have shown its potential to augment the ridge laterally [6,11]. When combined with bone grafts, the membrane enhances clinical attachment levels and reduces probing depths, making it a valuable tool in managing periodontitis [2,9].

In cases of peri-implantitis, CLBM has demonstrated its ability to regenerate bone around failing implants. Clinical studies have reported substantial bone gain and improved implant stability when CLBM is paired with xenografts, highlighting its utility in restoring compromised implant sites [8,10].

For ridge augmentation procedures, CLBM provides the necessary mechanical strength to stabilize graft materials and create adequate bone volume for implant placement. Deepika-Penmetsa et al. reported successful outcomes in lateral ridge augmentation, with the membrane ensuring graft stability and promoting long-term bone regeneration [6,7]. Also, a recent review highlighted the superior vertical bone gains achieved with cortical lamina compared to traditional membranes [12].

In sinus lift procedures, CLBM prevents graft displacement while promoting new bone formation. High success rates for implant placement have been documented in sites treated with CLBM, making it a reliable choice for sinus augmentation procedures [5,7]. CLBM holds several advantages over conventional membranes, which are listed in Table 1.

**Table 1:** Advantages of cortical laminate bone membrane over conventional membranes

Cortical laminar bone membrane	Conventional membrane
Superior mechanical stability [6-8]	Inferior mechanical stability (e.g., collagen membranes)
Promotes bone formation through its osteoconductive surface [1,3,4]	No osteoconductive surface (e.g., synthetic membranes)
Enhanced integration with host tissues [7,9]	Lacks biological compatibility (e.g., titanium-reinforced membranes)
No requirement of additional surgery for retrieval of membrane [13-15]	Requires additional surgery for retrieval (e.g., expanded polytetrafluoroethylene)

A list of published literature on cortical laminate bone membrane is shown in Table 2.

**Table 2:** Review of recent literature published related to cortical laminate bone membrane GBR: guided bone regeneration

Author(s)	Year	Key findings relevant to the review
Debortoli et al. [10]	2024	Cortical xenogeneic membranes provide stability and effective integration for GBR, demonstrating reliable performance in retrospective studies.
Takallu et al. [15]	2024	Antimicrobial enhancements in resorbable membranes improve their effectiveness in treating periodontitis and preventing infection during GBR procedures.
Yang et al. [16]	2023	The Yoda1 bilayer membrane enhances osteointegration and angiogenesis, accelerating bone regeneration in GBR.
Rossi et al. [9]	2023	Xenogenic cortical bone sheets act as a stable scaffold, showing effective outcomes in cases of severe bone atrophy and soft tissue regeneration.
Alqahtani et al. [1]	2023	Resorbable membranes are preferred due to their ease of use and reduced need for a second surgery. Advances in materials such as collagen improve biocompatibility and clinical outcomes.
Das et al. [2]	2022	Resorbable membranes demonstrate clinical stability and effectiveness in treating periodontal defects, with significant improvement in intrabony defect resolution.
Tumedei et al. [5]	2022	Cortical laminar membranes offer structural support and promote guided bone regeneration, showing promising biological integration over decades of use.
Rossi et al. [7]	2022	Bone lamina membranes facilitate guided bone and tissue regeneration, offering long-term stability and functional outcomes in complex cases.
Zhang et al. [12]	2022	Resorbable membranes perform comparably to non-resorbable ones in vertical bone regeneration, offering simplicity in handling and avoiding second-stage removal surgeries.
Schuh et al. [8]	2021	Porcine collagenated cortical bone lamina supports multilayer techniques in GBR, with effective results in immediate implantation and esthetic area reconstruction.
Tay et al. [17]	2020	Cortical laminar bone membranes, while providing structural support and stability, are susceptible to exposure-related issues that may lead to bacterial contamination and impaired bone regeneration. Proper surgical technique and soft tissue management are emphasized to mitigate these risks.
Khojasteh et al. [4]	2017	Resorbable membranes provide effective barrier functions, although their mechanical stability may be inferior to non-resorbable alternatives in larger defects.
Deepika-Penmetsa et al. [6]	2017	Cortical laminar membranes provide robust structural support for ridge augmentation, ensuring space maintenance and successful outcomes in lateral ridge regeneration.
Cucchi et al. [14]	2017	Resorbable membranes show lower complication rates but may achieve slightly less vertical bone gain compared to titanium meshes and non-resorbable membranes.
Wessing et al. [18]	2017	A novel non-cross-linked collagen membrane showed effective outcomes in GBR, particularly at implant sites with dehiscence, providing stability and biocompatibility.

Challenges and limitations

The rigidity of CLBM is advantageous for maintaining space, but at the same time, it poses a challenge to adapt the membrane to irregular or complex defects such as morphologies [6,8]. Unlike more flexible collagen-based membranes, CLBM requires precision in trimming and shaping to ensure optimal coverage of the defect. This inflexibility may increase surgical time and the potential for errors during placement. The use of cortical lamina requires precise handling and customization, making it technique-sensitive [15,16]. Deepika-Penmetsa et al. emphasized that achieving proper adaptation demands advanced surgical expertise, which may limit its use among less experienced clinicians or in high-stress clinical scenarios [6]. Due to its slower degradation rate, CLBM is prone to exposure if not adequately covered by soft tissue. Proper flap management is critical to minimizing this risk [9,10]. Membrane exposure not only compromises the regenerative outcome by increasing the risk of infection but can also necessitate additional surgical interventions to address complications [15]. Rossi et al. noted that poor flap management or insufficient soft tissue coverage could lead to exposure, particularly in cases involving thin gingival biotypes or significant defect dimensions [9]. This highlights the need for careful case selection and advanced soft tissue management techniques. The production of CLBM involves stringent processing to remove antigenic proteins while preserving its osteoconductive properties. This complex manufacturing process contributes to the high cost of the material, limiting its availability and adoption, particularly in resource-constrained settings [1,10]. Tumedei et al. pointed out that these costs could present a barrier to widespread clinical use, particularly when compared to more economical alternatives such as resorbable collagen membranes [5]. As cost efficiency becomes an increasingly important consideration in healthcare, this limitation highlights the need for continued innovation to make CLBM more accessible.

Although numerous studies have demonstrated the short- to mid-term success of CLBM in regenerative procedures, there is a relative lack of long-term clinical trials comparing its outcomes with traditional membranes or other advanced biomaterials. As Schuh et al. noted, long-term follow-up studies are essential to assess the durability and predictability of CLBM in diverse clinical contexts [8]. The absence of such data makes it challenging for clinicians to fully evaluate its advantages and risks over extended periods.

Future perspective

To expand the use of CLBM, ongoing research aims to enhance its handling properties, reduce production costs, and enhance antimicrobial properties. Additionally, incorporating bioactive molecules or growth factors could further enhance its regenerative potential. Long-term clinical trials and comparative studies are essential to validate its superiority over other advanced biomaterials.

## Conclusions

CLBM represents a significant step forward in regenerative dentistry. With their unique combination of mechanical stability, biocompatibility, and osteoconductive properties, CLBM addresses many of the limitations associated with traditional membranes. While challenges such as handling difficulties and costs remain, the transformative potential of CLBM positions it as a cornerstone in modern regenerative techniques. Ongoing innovations and research will continue to refine its applications, ensuring its role in achieving predictable and successful clinical outcomes.
